# Effect of intermediate care on mortality following emergency abdominal surgery. The InCare trial: study protocol, rationale and feasibility of a randomised multicentre trial

**DOI:** 10.1186/1745-6215-14-37

**Published:** 2013-02-02

**Authors:** Morten Vester-Andersen, Tina Waldau, Jørn Wetterslev, Morten Hylander Møller, Jacob Rosenberg, Lars Nannestad Jørgensen, Inger Gillesberg, Henrik Loft Jakobsen, Egon Godthåb Hansen, Lone Musaeus Poulsen, Jan Skovdal, Ellen Kristine Søgaard, Morten Bestle, Jesper Vilandt, Iben Rosenberg, Rasmus Ehrenfried Berthelsen, Jens Pedersen, Mogens Rørbæk Madsen, Thomas Feurstein, Malene Just Busse, Johnny D H Andersen, Christian Maschmann, Morten Rasmussen, Christian Jessen, Lasse Bugge, Helle Ørding, Ann Merete Møller

**Affiliations:** 1 Department of Anaesthesiology and Intensive Care Medicine, Herlev Hospital, Copenhagen University, Herlev Ringvej 75, DK-2730, Herlev, Denmark; 2 Copenhagen Trial Unit, Centre for Clinical Intervention Research, Rigshospitalet, Copenhagen University Hospital, Copenhagen, Denmark; 3 Department of Anaesthesiology and Intensive Care Medicine, Copenhagen University Hospital Rigshospitalet, Copenhagen, Denmark; 4 Department of Surgery, Herlev Hospital, Copenhagen University, Herlev, Denmark; 5 Department of Surgery, Bispebjerg Hospital, Copenhagen University, Copenhagen, Denmark; 6 Department of Anaesthesiology, Koege Hospital, Copenhagen University, Koege, Denmark; 7 Department of Surgery, Koege Hospital, Copenhagen University, Koege, Denmark; 8 Department of Anaesthesiology, Hilleroed Hospital, Copenhagen University, Hilleroed, Denmark; 9 Department of Surgery, Hilleroed Hospital, Copenhagen University, Hilleroed, Denmark; 10 Department of Anaesthesiology, Herning Regional Hospital, Herning, Denmark; 11 Department of Surgery, Herning Regional Hospital, Herning, Denmark; 12 Department of Anaesthesiology, Hospital of Southern Jutland Aabenraa, Aabenraa, Denmark; 13 Department of Surgery, Hospital of Southern Jutland Aabenraa, Aabenraa, Denmark; 14 Department of Anaesthesiology, Bispebjerg Hospital, Copenhagen University, Copenhagen, Denmark; 15 Department of Anaesthesiology, Hospital Lillebaelt Vejle, Vejle, Denmark; 16 Department of Surgery, Hospital Lillebaelt Vejle, Vejle, Denmark

**Keywords:** Emergency, Surgery, APACHE II score, Intermediate care, High-dependency unit, Postoperative care, Clinical trial, Randomised, Mortality, Length of stay

## Abstract

**Background:**

Emergency abdominal surgery carries a 15% to 20% short-term mortality rate. Postoperative medical complications are strongly associated with increased mortality. Recent research suggests that timely recognition and effective management of complications may reduce mortality. The aim of the present trial is to evaluate the effect of postoperative intermediate care following emergency major abdominal surgery in high-risk patients.

**Methods and design:**

The InCare trial is a randomised, parallel-group, non-blinded clinical trial with 1:1 allocation. Patients undergoing emergency laparotomy or laparoscopic surgery with a perioperative Acute Physiology and Chronic Health Evaluation II score of 10 or above, who are ready to be transferred to the surgical ward within 24 h of surgery are allocated to either intermediate care for 48 h, or surgical ward care. The primary outcome measure is all-cause 30-day mortality. We aim to enrol 400 patients in seven Danish hospitals. The sample size allows us to detect or refute a 34% relative risk reduction of mortality with 80% power.

**Discussion:**

This trial evaluates the benefits and possible harm of intermediate care. The results may potentially influence the survival of many high-risk surgical patients. As a pioneer trial in the area, it will provide important data on the feasibility of future large-scale randomised clinical trials evaluating different levels of postoperative care.

**Trial registration:**

Clinicaltrials.gov identifier: NCT01209663

## Background

Worldwide an estimated 234 million surgical procedures are performed every year [1]. Overall, non-cardiac surgery carries low postoperative mortality rates of 1.4% to 1.9% [2-4]. However, this conceals the fact that a subgroup has a high risk of postoperative death. This high-risk group comprises patients often of advanced age with significant co-existent disease undergoing complex emergency surgery [4]. Emergency abdominal surgery carries a 15% to 20% short-term mortality rate [5-8], with cardiopulmonary complications and sepsis as the most frequent causes of death [9]. Postoperative medical complications are more important determinants of postoperative death than preoperative demographic characteristics, and intraoperative adverse events [8,10]. Recent research suggests that timely recognition, and effective management, of postoperative complications may reduce mortality [11]. Thus, early routine postoperative admission of high-risk non-cardiac surgical patients to intensive or intermediate care units could prove important. However, many countries have limited access to these facilities [4,12]. The majority of high-risk non-cardiac surgical patients are consequently treated on standard surgical wards with restricted resources for monitoring, and advanced treatment methods [4,13]. Intermediate care (IC) could be an appropriate level of care for stable postoperative patients with an *a priori* high risk of complications and death. IC is generally defined as a level of care intermediate between that provided by a general ward and an intensive care unit. An IC unit monitors and supports patients with, or likely to develop, acute (or acute on chronic) single organ failure [14-16]. The effect of postoperative IC compared to ward care on high-risk surgical patients’ outcome is only sparsely evaluated, and never in a randomised clinical trial [17-23] (Additional file 1: Search string).

The aim of the InCare trial is to evaluate the effect of postoperative IC following emergency major abdominal surgery in high-risk patients identified by an Acute Physiology and Chronic Health Evaluation (APACHE) II score of 10 or above. The APACHE II score is a well-established classification system of the severity of diseases used in intensive care units worldwide [24], which can be used on emergency major abdominal surgical patients as well [5,25-31]. Emergency major abdominal surgical patients with an APACHE II score of 10 to 11 and above have a 28% to 45% mortality rate compared to patients with an APACHE II score below 9 and 10 who evidence a mortality rate of 0% to 7% [27-31]. We hypothesise that postoperative IC will lead to a reduction in postoperative mortality by avoidance of, or timely recognition and effective management of, postoperative complications. Furthermore, we hypothesise that early admission to postoperative IC may reduce later admission to intensive care.

## Methods and design

### Trial design

The InCare trial is an ongoing multicentre, randomised, parallel-group, non-blinded clinical trial with 1:1 allocation. Emergency abdominal surgical patients are postoperatively allocated to either: (1) IC for 48 h; or (2) surgical ward care (standard treatment). The trial was initiated in October 2010, and we aim to enrol 400 patients.

### Setting

In Denmark all emergency surgical patients are treated in tax-financed public-healthcare centres. The total intensive and intermediate care availability in Denmark is 8.3 beds per 100,000 citizens in 2010, with seven intensive care beds per 100,000 citizens and 1.3 intermediate care beds per 100,000 citizens (unpublished data from The Danish Regions central office). In comparison the intensive care availability in the United States was 20.0 per 100,000 citizens, and in the United Kingdom it was 3.5 per 100,000 citizens in 2005 [32]. Currently seven Danish tertiary referral university affiliated public-healthcare centres are participating in the trial.

### Inclusion criteria

Patients are eligible for inclusion if: (1) they have undergone emergency gastrointestinal laparotomy or laparoscopic surgery; (2) they are ready to be transferred to the ward after a postoperative stay in a post-anaesthesia care unit or an intermediate/intensive care unit for <24 h; and (3) they have a perioperative APACHE II score of ≥10. We define emergency surgery as indicated surgery, which should be undertaken within 24 h.

Because of slower enrolment rate than anticipated, the steering committee have decided to include patients with Apache II scores of 10 and 11 although the original inclusion criteria was an Apache II score ≥12. This was initiated on 23 May 2012 after 192 enrolled patients. Patients with Apache II scores of 10 and 11 also have a high 30-day mortality and the potential to benefit from the IC intervention [27-31].

### Exclusion criteria

Exclusion criteria are as follows: (1) appendectomy; (2) laparoscopic cholecystectomy; (3) negative diagnostic laparoscopy; (4) intensive care not indicated (patients on palliative care, or with irreversible organ failure); (5) previous participation in the trial; (6) age <18 years; (7) trauma; and (8) no IC bed available.

### Recruitment, screening and enrolment

**Figure 1:**
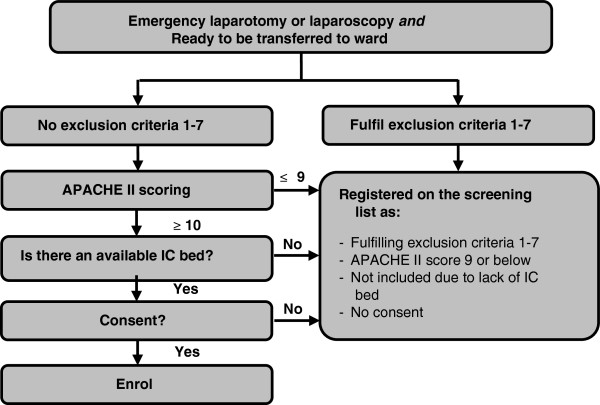
Screening flowchart.

We recruit patients postoperatively in the post-anaesthesia care unit and intensive/intermediate care unit. All adult patients who have undergone the relevant surgical procedures have a venous and arterial blood gas sample taken upon arrival from the operating theatre. Uniformity of trial site standard postoperative vital-sign monitoring is ensured, enabling calculation of the APACHE II score, when patients are ready to be transferred to the surgical ward in accordance with the trial site’s discharge criteria - local adjusted versions of the Danish national recommendations [33] (Additional file 2: National discharge recommendations). At this point of time the attending anaesthetist screens for trial eligibility using a standard screening form with flowcharts (Figure 1). If none of the exclusion criteria 1 to 7 apply, the APACHE II score is calculated from the worst parameter values measured 12 h preoperatively, peroperatively, and up to 12 h postoperatively. If there is missing data for the APACHE II score calculation, when the patient is transferable to the ward, current values are used. GCS score, blood pressure and heart rate recorded during anaesthesia are not included in the APACHE II score calculation. The APACHE II score is calculated electronically on the InCare trial website [34]. If the patient has an APACHE II score of ≥10 the anaesthetist checks that an IC bed can be established at the intermediate care, intensive care or post-anaesthesia care unit. If an IC bed is not available, the patient is registered as ‘not included due to lack of IC bed’ and transferred to the ward. If an IC bed is available, written consent is obtained from the patient or a legal representative, if the patient is incapable. If it is not possible to contact a legal representative within 2 h, the patient is included in the trial, and written consent is obtained as soon as possible.

### Randomisation

The patients are randomised using a telephone-based central interactive-voice-response system managed by the Copenhagen Trial Unit (CTU). The randomisation system is accessible around the clock. It ensures immediate computer-based allocation, and adequate allocation concealment. A computer-generated block randomisation, with the investigators unknown and varying block size is used. Stratification variables are: trial site; APACHE II score (10 to 14 or ≥15); and perforated viscera (yes *versus* no).

### Interventions

#### Intermediate care

IC is initiated as soon as the patient is allocated, and continues for at least 48 h. The IC bed is situated at an intermediate care, an intensive care or a post-anaesthesia care unit, which can provide the outlined intervention (Table 1). IC is defined as a minimal monitoring level and maximal treatment level. The minimal monitoring level is applied when the patient’s vital signs are stable. If the patient deteriorates, the level of monitoring and treatment is increased as indicated. When the maximal level of treatment defined in the IC is exceeded, that is when there is a need for invasive ventilation, emergency dialysis, parallel sympathomimetic drug infusion or invasive arterial-blood pressure monitoring, the patient is categorised as an intensive care patient. However, the patient remains in the trial for observation of the outcome measures. Surgeons and intensivists make compulsory protocol-based rounds on a daily basis using a standard form (Additional file 3: Surgeon - protocol-based round and Additional file 4: Intensivist - protocol-based round). All medical treatment and investigations are made on medical indication alone, not determined by the trial-protocol. Forty-eight hours after randomisation, or at morning handover if the patient is included at night, re-evaluation is made. If patients are stable, and local discharge criteria are met, they can be transferred to the surgical ward.

**Table 1:** Definition of intermediate care

**Observation**	**Minimal monitoring level^a^**	**Treatments goals^b^**	**Comments**
Level of consciousness	Every 8 h	GCS: 15	
Respiratory rate	Every second hour	RR: 10 to 20	If the patient has stable vital signs the RR is not measured during nights
Oxygenation	Continuous pulse oximetry	SpO_2_ ≥94%	Continuous pulse oximetry when the patient is supine or sitting in a chair. Discontinued during mobilisation
Blood pressure	Every second hour	MAP: 65 to 110 mmHg	If the patient has stable vital signs the MAP is not measured during nights
Heart rate	Continuous ECG monitoring	HR: 50 to 100	Continuous ECG when the patient is supine or sitting in chair. Discontinued during mobilisation. Diagnostic ECG on indication. If arrhythmia or ischaemia is detected the treatment goals are adjusted to current recommendations
		No ischemia	
Diuresis	Every hour	≥0.5 mL/kg/h	During mobilisation the diuresis is summed every third hour
Temperature	Every 8 h	36°C to 38°C	
Pain Visual Assessment Score	Every 8 h	VAS: 0 to 2 during rest	No VAS scoring during sleep
		Epidural: Able to move both legs	
Central venous pressure	Every 8 h	8 to 12 mmHg	CVP and S _c_VO_2_ is only registered if there is a central venous catheter in place. The central venous catheter is removed when possible
Central venous oxygen saturation	Every 8 h	SpO_2_ ≥ 70%	
Standard blood samples	Every 24 h	Within normal reference values	Hgb ≥4.5 mmol/L
			Hgb ≥6.0 during sepsis or heart disease
**Treatment(if needed)**	**Maximal treatment level**	**Treatments goals**	**Comments**
Single sympathomimetic drug support	Continuously	MAP: ≥65 mmHg	
		Diuresis: ≥0.5 mL/kg/h	
Oxygen therapy on open systems	Continuously	SpO_2_ ≥94%	Unless contraindicated, oxygen therapy is discontinued when oxygenation is ≥94% without oxygen therapy. During nights: minimum 2 L supplemental oxygen is given
Positive Expiratory Pressure (PEP) therapy	Assistance to PEP therapy: once per hour	SpO_2_ ≥94%	If the patient does not need assistance with PEP therapy, guidance in self-administration of PEP therapy must be available
Non-invasive ventilation	Continuously	Normocapnia and normoxic	
Volume / Fluid therapy	Continuously	MAP: ≥65 mmHg	Fluid balance: Evaluation frequency in accordance with monitoring level and vital signs
		Diuresis: ≥0.5 mL/kg/h	
		S _c_VO_2_ ≥70%	
		CVP: 8 to 12 mmHg	

During evening and night shifts: Staff specialist in anaesthetist/intensive care medicine on in-house duty and staff specialist in surgery on call.

^a^The minimal monitoring level is exceeded when necessary (for example, deterioration).

^b^All treatment goals are adjusted to the individual patient’s co-morbidities, physiological status and in the event of complications in agreement with current recommendations (for example, troponin T/I is measured when cardiac ischaemia is suspected).

#### Ward care

Patients allocated to ward care (WC) are transferred to the surgical ward with a protocol-based discharge note using a standard form (Additional file 5: Anaesthetist - discharge note). Patients are thoroughly evaluated before transfer, and a written plan of treatment and monitoring for the first 24 h in the ward is outlined in the medical chart. Otherwise, surgical ward treatment is as standard for the individual ward. In general, the participating surgical wards have the resources and facilities to meet the monitoring and treatment levels outlined in Table 2. This level can only be exceeded for a short time, and it is not possible to initiate continuous monitoring of vital signs. If patients deteriorate they are transferred to an intermediate care, or intensive care unit to be treated appropriately.

**Table 2:** Surgical ward care: an overview of facilities

**Observation**	**Monitoring level**	**Treatments goals^a^**	**Comments**
Level of consciousness	Every 8 h	GCS: 15	
Respiratory rate	Every 8 h	RR: 10 to 20	
Oxygenation	Every 8 h	SpO_2_ ≥94%	Continuous pulse oximetry is not available
Blood pressure	Every 8 h	MAP: 65 to 110 mmHg	
Heart rate	Every 8 h	HR: 50 to 100	Continuous ECG is not available. Diagnostic ECG on indication. If arrhythmia or ischaemia is detected the treatment goals are adjusted to current recommendations
		No ischaemia	
Diuresis	Every 8 h	≥0.5 mL/kg/h	
Temperature	Every 8 h	36°C to 38°C	
Pain Visual Assessment Score	Every 8 h	VAS: 0 to 2 during rest Epidural: Able to move both legs	
Central venous pressure	Not available		
Central venous oxygen saturation	Not available		
Standard blood samples	Every 24 h	Within normal reference values	Hgb ≥4.5 mmol/L
			Hgb ≥6.0 during sepsis or heart disease
**Treatment(if needed)**	**Maximal treatment level**	**Treatments goals**	**Comments**
Infusion of sympathomimetic drugs	Not available		
Oxygen therapy on open air systems	Continuously	SpO_2_ ≥94%	Unless contraindicated. Oxygen therapy is discontinued when oxygenation is above ≥94% without oxygen therapy. During nights: minimum 2 L supplemental oxygen is given
Positive Expiratory Pressure (PEP) therapy	Assistance to PEP therapy: every fourth hour during day and evening shift	SpO_2_ ≥94%	If the patient does not need assistance with PEP therapy, guidance in self-administration of PEP therapy is available
Non-invasive ventilation	Not available		
Volume / Fluid therapy	Continuously	Systolic blood pressure: ≥100 mmHg	Fluid balance: Evaluation frequency in accordance with monitoring level and vital signs
		Diuresis: ≥12 mL/kg/day	

During evening and night shifts: Resident in surgery on in-house duty and staff specialist in surgery on call. Staff specialist in anaesthesiology/intensive care medicine on call from in-house duty.

^a^All treatment goals are adjusted to the individual patient’s co-morbidities, physiological status and in the event of complications in agreement with current recommendations.

### Baseline data

After inclusion we register the following demographic characteristics: dementia; previous stroke; chronic obstructive pulmonary disease; previous pulmonary embolism; previous myocardial infarction; chronic kidney dialysis; cancer; tobacco habit; alcohol consumption; assistance with personal hygiene; The American Society of Anaesthesiologists’ Physical Status Classification (ASA) score, and the postoperative sepsis score: grade 0, no sepsis and no systemic-inflammatory-response-syndrome (SIRS); grade 1, SIRS; grade 2, sepsis; grade 3, severe sepsis; and grade 4, septic shock [35]. We collect the following perioperative data: nature and duration of surgery; method of anaesthesia; location and duration of post-anaesthesia care; infusion of sympathomimetic drug (yes/no); blood loss; volume of crystalloid and/or colloid infusion; and infusion of blood products (type and number of units).

### Intervention period data

We measure compliance with the trial protocol by registering the frequency of observations of the measures listed in Table 3, made during the 48 h intervention period. Additionally, we register the following measures regarding level of treatment and monitoring in both groups: early-warning-score system [36] monitoring for >24 h (yes/no); nutrition initiated within 24 h (yes/no); mobilisation within 24 h of surgery (yes/no; level); mobilisation within 48 h of surgery (yes/no; level); intensivist or anaesthetist evaluation (number; specialist/non-specialist), and surgeon evaluation (number; specialist/non-specialist). Additionally, we register the use of diagnostic imaging: chest X-ray; lung scintigraphy; and echocardiography. In the IC group we register reason for: premature or late transfer to the surgical ward (respectively before or after 48 h intervention); ‘step-up’ to an intensive care bed; the Simplified Acute Physiology Score (SAPS) II [37]; the Sequential Organ Failure Assessment (SOFA) score (day 1 and 2) [38]; and the sepsis score (day 2).

**Table 3:** Compliance with trial protocol

**Measures**	**IC Group (day 2)^a^**	**WC Group (day 14)^a^**
**Actual monitoring level**		
Level of consciousness (number of registrations)	x	x
Respiratory rate (number of registrations)	x	x
Continuous pulse oximetry (yes/no)	x	x
Blood pressure (number of registrations)	x	x
Continuous ECG monitoring (yes/no)	x	x
24-h diuresis (number of registrations)	x	x
Hourly diuresis registration for >24 h (yes/no)	x	x
Temperature (number of registrations)	x	x
Pain Visual Assessment Score (no. of registrations)	x	x
Central venous pressure (number of registrations)	x	x
Central venous oxygen saturation (number of samples)	x	x
Standard blood samples (number of samples)	x	x
**Treatment level**		
Infusion of sympathomimetic drugs (yes/no)	x	x
Parallel infusion of sympathomimetic drugs (yes/no)	x	x
>2 L supplemental oxygen during nights (yes/no)	x	x
Assistance to PEP therapy (number of treatments)	x	x
Non-invasive ventilation (yes/no)	x	x
Invasive ventilation (yes/no)	x	x
Emergency dialysis (yes/no)	x	x
24-h fluid balance calculation (number of registrations)	x	x
Protocol-based discharge by anaesthetist (yes/no)^b^		x
Protocol-based round by intensivist (number)	x	
Protocol-based round by surgeon (number)	x	
Patient location (hours)^c^	x	x

Data stem from medical charts, nurse charts and observation charts used in the 48-h intervention period.

^a^Timing of registration of compliance to protocol in the case report form.

^b^Registered by the anaesthetist writing the discharge note and checked at day 14.

^c^Post-anaesthesia care unit; intermediate care bed; intensive care bed; surgical ward; medical ward; and/or coronary care unit.

### Follow-up

Mortality data will be retrieved from The Danish Civil Registration System (CRS) [39]. The CRS contains mortality data on all Danish citizens, through a unique personal identification number. The retrieval from CRS will be made by an independent data manager at Copenhagen Trial Unit at interim analysis and at day 30 after the last patient has been enrolled. The secondary outcome measures are registered from the medical chart at day 30 postoperatively, and from the National Patient Registry (NPR), which contains data on hospitalisation of all Danish citizens [40]. Furthermore, return to operating theatre, and postoperative medical and surgical complications requiring treatment within 14 days of randomisation, are retrieved from the medical chart at day 30.

### Outcome measures

The primary outcome measure is all-cause 30-day mortality.

Secondary outcome measures are: (1) all-cause mortality within the total observation time, measured 30 days after the last included patient; (2) admission to an intensive care unit, and duration thereof, within 30 days of randomisation; and (3) duration of hospitalisation postoperatively (days).

### Data collection and trial conduct

Data are collected on printed case report forms, on which a unique barcode number is printed to eliminate the possibility of duplication of the case report forms. Case report forms are scanned into the database using the Verity Teleform® system (Verity, Sunnyvale, CA, USA) managed by Copenhagen Trial unit. Mortality data is extracted from The CRS. The Steering Committee will not have access to the dataset until the trial has been completed. The InCare trial is conducted in compliance with the Helsinki Declaration, and is approved by the Copenhagen Capital Region Ethical board (H-3-2010-010) and the Danish Data Protection Agency (HEH.afd.I.750.16-18). The trial is registered at clinicaltrials.gov (NCT01209663), and conducted and monitored in accordance with the ICH-GCP guidelines. Case report forms are checked for validity and internal consistency through trial site visits by Good Clinical Practice (GCP) monitors, who checks source data in approximately 10% of the case report forms selected at random. The trial is designed in compliance with the Consort Statement, and the two extensions: ‘Improving reporting of pragmatic trials’ and ‘Extending the consort statement to randomised trials of nonpharmacologic trials’ [41-43].

### Interim analyses

We have established an independent Data Monitoring Committee (DMC) to evaluate safety and efficacy on scheduled interim analyses of 30-day mortality data. The monitoring plan is based on the modified Haybittle-Peto boundaries for stopping trials after interim analyses in the second half of the inclusion period [44,45]. The mortality data are presented to the DMC under a blinded code for allocation group. The first interim analysis will be conducted when 30-day mortality data of 200 trial participants have been obtained, and/or 75 deaths have been documented during the trial. If the first interim analysis of 30-day mortality data is significant (*P* <0.001) for benefit or harm from the intervention, a second interim analysis will be made when 30-day mortality data of 300 trial participants have been obtained and/or an additional 25 deaths have been documented. If the second interim analysis is also statistically significant (*P* <0.001) for benefit or harm from the intervention the DMC can advise the steering committee to stop the trial.

### Statistical analysis

The outcome measures will be analysed for all randomised patients in a modified intention-to-treat analysis leading to the primary results of the trial [46]. Patients with the following major protocol violations will not be included in a per-protocol analysis: not fulfilling an inclusion criterion; fulfilling an exclusion criterion; IC for <48 h because of early discharge to the ward. The primary outcome measure, 30-day mortality, will be analysed with an unadjusted univariate logistic regression, and a multivariate logistic regression analysis will be made adjusting for stratification variables: trial-site, APACHE II score (10 to 14 or ≥15), and perforated viscera (yes *versus* no); and design variables: age, ASA score (1 to 2 or ≥3), cancer, and nature of surgery (+/− re-operation). The secondary outcome measure, survival within the total observation time, will be analysed with an unadjusted Cox regression analysis, and a Cox regression analysis adjusted for the above-mentioned stratification variables and design variables [47]. Survival within the total observation time will be illustrated with Kaplan-Meier estimates. Fisher’s exact or chi-squared tests will be used, depending on values, to analyse the differences of frequencies of registered complications in the two groups. Mortality data will be retrieved on patients registered as ‘not included in the trial due to lack of IC bed’. The data on these patients will be compared with that from the WC group, then included with the WC group data and compared with the IC group. Data on a semi-quantitative scale will be analysed using the Mann–Whitney test. Double sided *P* values <0.05 will be considered statistically significant.

### Sample size

Emergency major abdominal surgical patients with an APACHE II score ≥10/11 have a 28% to 45% mortality rate [27-31]. We assume that patients with an APACHE II score of ≥10 have a 30-day mortality of 38%. We aim to confirm or reject an intervention effect of 13% absolute risk reduction in mortality (a relative risk reduction of 34%), in accordance with the difference between group mortality detected in our recent observational study of optimised perioperative care in perforated peptic ulcer patients [48]. The sample size with a type 1 error risk of 5%, and a type 2 error risk of 20% (80% power), was estimated to 200 patients in each group [49].

### Ethical considerations

Randomised trials in presumed optimised health-care protocols are associated with unique ethical problems, especially when mortality is the outcome measure. Patients often intuitively find the IC appealing when the interventions are outlined for them, but IC is only available by chance if they consent to participate in the trial. In this design it is of utmost importance to consider that the effect of postoperative intermediate care, with a package of possible interventions, remains to be proven. Furthermore, participants are exclusively enrolled postoperatively when they fulfill the current discharge criteria to be transferred to the surgical ward, which ensures that the control group is given the best current available standard treatment.

In the InCare trial, all diagnostic tests and interventions in the control and intervention group are initiated by the attending doctors, thereby reflecting their medical judgment, and not alone the trial protocol. We are thus only evaluating the effect of the extra monitoring, extra specialist attention and patient placement in IC or WC with differences in routine, resources, and capability.

### Trial management

The InCare trial is investigator initiated, controlled by a steering committee including anaesthetists, intensivists and surgeons, all with solid research experience. The committee is responsible for the planning, design and conduct of the trial. At each trial site two or three site investigators with at least one surgeon and one intensivist/anaesthetist manage the local implementation. This includes teaching and supervising of staff, daily management, and data collection with support from the principal coordinating investigator. Protocol observance is stimulated by repeated staff educational sessions on patient enrolment, and execution of the trial interventions by the site investigators and the coordinating investigator. Additionally, intervention period data are checked to monitor adherence to protocol, thereby giving feedback to staff. A monthly newsletter with trial updates is sent to the involved parties, and published on the trial website.

## Discussion

### Trial rationale

#### Participants

Our recent findings in emergency surgical patients with perforated ulcer [48] suggested that we should investigate the effects of IC in emergency major abdominal surgical patients with an APACHE II score ≥10. The APACHE II index is well-known to anaesthetists responsible for patient enrolment, and it reflects the presence of perioperative sepsis, cardiovascular and respiratory failure.

#### Interventions

The level of care in the IC group is based on national and international definitions, descriptions, surveys and recommendations which may increase the external validity [15,50-53].

We chose an IC admission duration of 48 h influenced by the findings of Garmil *et al*. [54] that 80% of patients dying or disabled in hospital began to deteriorate within 24 h postoperatively, and of Jhanji *et al*. [13] that surgical ward admission was approximately 48 h prior to the necessary postoperative intensive care unit admission. Furthermore, this duration of the IC intervention was conceived realistic in that it could be accepted by the participating trial sites.

Daily protocol-based rounds are made in the IC group to ensure uniform evaluation at the participating trial sites, thus improving internal validity. The rounds made by surgeons and intensivists, are aimed at optimising pain relief, cardiopulmonary function, fluid balance, nutrition, mobilisation and early detection of complications.

The WC group receives ward care according to local standards. We choose not to reinforce a treatment and monitoring protocol in the WC group, as we wish to compare IC with standard care at the participating trial sites. With trial site stratified randomisation, we aim to adjust for possible differences in standard care at the wards. Additionally, we register intervention period data on level of treatment and monitoring as in the IC group, which enable us to report the actual level of treatment and monitoring in the wards.

In the WC group, intervention period data collection is made on day 14, because data collection on day 2 would equate with an extra clinical evaluation and the possible initiation of treatment, which could differ from current standard care.

### Previous research

The effect of postoperative IC is unresolved in high-evidence studies. Previous studies of IC have been non-randomised, observational or descriptive, often with contradictory results [17-23]. Davies *et al*. and Bellomo *et al*. [18,19] found no reduction in postoperative in-hospital mortality or morbidity following introduction of an IC unit at their facilities. Both studies reported a change in surgical intake tending more to emergency surgery after introduction of the IC unit. This may explain the lack of effect in their unadjusted analyses. Turner *et al*. [20] found an excess crude mortality rate in patients not admitted to IC units despite requests from the attending surgeon or anaesthetist. In a similar design McIlroy *et al*. [21] concluded that IC may lead to longer hospital stay, but no effect on 30-day mortality or all-cause morbidity could be shown. Jones *et al*. [22] compared two hospitals (one with, and one without, an IC unit). This study suggested that postoperative IC might reduce cardiopulmonary complications after major abdominal surgery, when risk adjusting with Physiologic and Operative Severity Score for the enumeration of mortality and morbidity (POSSUM score). No effect on mortality or length of hospital stay could be shown. Swart *et al*. [23] compared postoperative intensive care with surgical ward care in colorectal surgical patients with a preoperative anaerobic threshold <11 mL oxygen/ kg/min. They found that there was significant decrease in cardiac adverse events in the intensive care patients, but no effect on in-hospital mortality, or length of hospital stay could be shown.

Common in the previous research is a high risk of bias as confounding by indication and selection bias, and thereby unbalanced number of risk patients in the compared groups is imminent. Furthermore, the cited studies are characterised by limited power to show effect on mortality, because they predominantly investigate patients undergoing scheduled surgical procedures which have much lower mortality than emergency abdominal surgical procedures.

### Strengths

The InCare trial is the first randomised clinical trial to evaluate the effect of postoperative intermediate care. The design will provide evidence with low risk of bias compared with previous studies.

We have thoroughly described the complex intervention in the IC group and the compliance with protocol is rigorously registered to improve internal validity, and transparent reporting of the trial. IC is evaluated in high-risk emergency abdominal surgical patients with high mortality. This enables us to report all-cause mortality, in a relevant patient population, which we believe is the most relevant primary outcome measure when evaluating IC. We have chosen not to report postoperative complications as an outcome measure. Our hypothesis is that the IC will lead to avoidance or earlier recognition of postoperative complications. Reporting complications when evaluating IC compared with WC can be misleading in that IC may reduce complications because of early postoperative optimisation, or show an increase in registered complications because of more intensive controls, for example, X-rays/atelectasis, which might not be detected on the ward and spontaneously resolve. Given that the IC both can lead to a decrease (avoidance of complications) or an increase (recognition of complications) in the registered postoperative complication rate, appropriate conclusions on these outcomes will be difficult in the case of no mortality effect because of type II error. Additionally, if complications were to be reported as an outcome measure, a detailed prospective bedside registration during the first 14 to 30 days after surgery would be necessary. These investigator follow-up visits would be equivalent to extra clinical evaluations of the patients, which might well entail change in the current standard care in the IC and WC groups. This could potentially jeopardise the external validity of the primary outcome measure and conceal a beneficial effect of IC on mortality.

### Limitations

In this pragmatic clinical trial some important limitations must be noted. Unfortunately, it is not possible to blind the involved healthcare personnel, or the participants, in this trial, and outcome assessment is only partially blinded. Given that the data on mortality are retrieved from The Danish Civil Registration System the risk of detection bias is minimal compared to, for example, an assessment of postoperative complications as an outcome. The lack of blinding, and the fact that the same surgeons at the same hospital treat both the IC and WC group may insinuate a learning bias possibly concealing a beneficial effect of the IC. In the InCare trial the WC group receives local standard care, which is not rigorously defined by the protocol. This is a limitation. Our efforts to register intervention period data will provide important information on the actual monitoring and treatment given to the WC group, and will also expose any learning bias in the WC group.

The intervention is complex, and the involvement of several trial sites entails the risk that not all elements of intervention are uniformly applied. Nevertheless, these deviations may reflect clinical practice, and the transparent reporting will give ground for a fair interpretation of the trial result. Finally, as this trial is the first of its kind, the sample size calculation is based on estimates, which entails a risk of type II error, thus not detecting a smaller intervention effects than the anticipated relative risk reduction of 34% used in our sample size estimation.

## Conclusion

The InCare trial is the first randomised clinical trial that aims to contribute to answer the important research question: should high-risk surgical patients receive postoperative intermediate care. The results may potentially influence the survival of many high-risk surgical patients. As a pioneer trial in the area, it will provide important data on the feasibility of a future large randomised clinical trial, which may definitively settle the question.

### Trial status

All Danish public-healthcare centres that receive emergency major abdominal surgical patients were asked to participate in the trial (*n* = 23). The reasons for declining to participate were: limited intensive/intermediate care facilities (*n* = 8); high-risk surgical patients treated >24 h postoperatively in a post-anaesthesia care unit or an intermediate/intensive care unit (*n* = 3); conflicting research projects (*n* = 2); lack of resources to conduct more than one multicentre clinical trial (*n* = 1), and no answer after multiple contacts (*n* = 2).

Seven centres thus currently participate in the trial and have started enrolment: Herlev Hospital, 4 October 2010 (*n* = 80); Koege Hospital, 11 October 2010 (*n* = 35); Hilleroed Hospital, 15 November 2010 (*n* = 36); Herning Hospital, 2 May 2011 (*n* = 19); Aabenraa Hospital, 1 July 2011 (*n* = 2); Bispebjerg Hospital, 1 December 2011 (*n* = 17); and Vejle Hospital, 1 February 2012 (*n* = 10). To date (1 June 2012) 199 patients have been enrolled.

## Abbreviations

APACHE: Acute Physiology and Chronic Health Evaluation; ASA: American Society of Anaesthesiologists physical status classification score; BP: Blood pressure; CENTRAL: Cochran Central Register of Controlled Trials; CPAP: Continuous positive airway pressure; CRP: C-reactive protein; CRS: The Civil Registration System; CTU: Copenhagen Trial Unit; CVP: Central venous pressure; DMC: Data monitoring committee; DVT: Deep venous thrombosis; ECG: Electrocardiogram; GCS: Glasgow Coma Scale; HGB: Haemoglobin; HR: Heart rate; IC: Intermediate care; ICH-GCP: International Conference on Harmonization Good Clinical Practice; MAP: Mean arterial pressure; NPR: National Patient Registry; PEP: Positive expiratory pressure; POSSUM: Physiologic and Operative Severity score for the enumeration of mortality and morbidity; RBC: Red blood count; RR: Respiratory rate; SAPS II: Simplified Acute Physiology Score II; S _c_VO_2_: Central venous saturation; SIRS: Systemic-Inflammatory-Response-Syndrome; SOFA: Sequential Organ Failure Assessment score; SpO_2_: Oxygen saturation; TP: Temperature; VAS: Pain Visual Assessment Score; WC: Ward care; WBC: White blood count.

## Competing interests

The authors declare that they have no competing interests.

## Authors’ contributions

Each author has made substantial intellectual contributions to the conception, design or implementation of The InCare trial and has been involved in the critical revision of the manuscript. All authors have approved the final manuscript for submission. MVA is the principal coordinating investigator. MVA, TW, JW, MHM, JR, LNJ and AMM are members of the steering committee. TW, IG, HLJ, EGH, LMP, JS, EKS, MB, JV, IR, REB, JP, MRM, TF, MJB, JA, CM, MR, CJ, LB and HØ are principal site investigators and have coordinated the implementation, enrolment, intervention or follow-up at their centre. AMM is the sponsor.

## Supplementary Material

Additional file 1 Search string. Click here for file

Additional file 2 National discharge recommendations. Click here for file

Additional file 3 Surgeon - protocol-based round. Click here for file

Additional file 4 Intensivist - protocol-based round. Click here for file

Additional file 5 Anaesthetist - discharge note. Click here for file
