# Supplementary material for: Effect of intermediate care on mortality following emergency abdominal surgery. The InCare trial: study protocol, rationale and feasibility of a randomised multicentre trial
Source: Trials. 2013 Feb 2;14:37. doi: 10.1186/1745-6215-14-37 (PMC3575365; doi:10.1186/1745-6215-14-37)
Supplement: Additional file 1 — Search string. [file 1745-6215-14-37-S1.pdf]

A non-language restricted search for randomised trials evaluating the effect of postoperative intermediate care as compared to ward care was done in MEDLINE, EMBASE and Cochrane Library (including CENTRAL). The last search was performed in marts 2012.

Database: Ovid MEDLINE(R) <1948 to 2012 Week 5>
Search Strategy:

- #1 ((care or area or unit or therapy) adj3 (((scheduled or postoperative) and (intensive or critical)) or intermediate or high?dependency)).ti,ab. or exp Intensive Care Units/ or Intensive Care/ or Critical Care/ (84504)
- #2 ((postoperative* or ward) adj3 care).mp. or exp Postoperative Care/ (53098)
- #3 #1 and #2 (3502 references)

Database: Embase <1980 to 2012 Week 10>
Search Strategy:

- #1 ((care or area or unit or therapy) adj3 (((scheduled or postoperative) and (intensive or critical)) or intermediate or high?dependency)).ti,ab. or exp intensive care unit/ or intensive care/ (127734)
- #2 ((postoperative* or ward) adj3 care).mp. or exp postoperative care/ (62984)
- #3 #1 and #2 (4105 references)

Database: Cochran Library <Searched marts 2012>
Search strategy

- #1 MeSH descriptor Intensive Care Units explode all trees (2042)
- #2 MeSH descriptor Intensive Care explode all trees (971)
- #3 MeSH descriptor Critical Care explode all trees (1569)
- #4 ((care or area or unit or therapy) near (((scheduled or postoperative) and (intensive or critical)) or intermediate or high?dependency)):ti,ab (1329)
- #5 (#1 OR #2 OR #3 OR #4) (4454)
- #6 MeSH descriptor Postoperative Care explode all trees (3438)
- #7 ((postoperative* or ward) near care) (6554)
- #8 (#6 OR #7) (6554)
- #9 (#5 AND #8) (627 references)
