# Supplementary material for: Effect of intermediate care on mortality following emergency abdominal surgery. The InCare trial: study protocol, rationale and feasibility of a randomised multicentre trial
Source: Trials. 2013 Feb 2;14:37. doi: 10.1186/1745-6215-14-37 (PMC3575365; doi:10.1186/1745-6215-14-37)
Supplement: Additional file 2 — National discharge recommendations. [file 1745-6215-14-37-S2.pdf]

Danish recommendations for choice of discharge criteria from anaesthesiology department to the surgical ward after anaesthesia, version 5.0

Proposal for scoring system

Measures	Points	Criteria	Arrival point score	10 min before transfer	Discharge point score
Sedation	3<br>2<br>1<br>0	Asleep constantly, can not be awakened<br>Asleep constantly, awakened by physical stimuli<br>Sleepy, aroused verbally<br>Awake, alert			
Respiratory<br>Respirations min^{-1}	3<br>2<br>1<br>0	Periods of apnea or obstruction<br>RR < 10 or RR > 30<br>Snoring 10 < RR < 30<br>Normal RR ≥ 10			
Oxygenation<br>Without oxygen for 10 min.	3<br>2<br>1<br>0	< 85%<br>85 ≤ SpO_2 < 90<br>90 ≤ SpO_2 < 94<br>≥ 94			
Blood pressure<br>Systolisk BP without sympatho-mimetica (mmHg)	3<br>2<br>1<br>0	< 80<br>80 ≤ BP < 90 or BP > 220<br>90 ≤ BP < 100<br>≥ 100			
Heart rate<br>Beats min^{-1}	3<br>2<br>1<br>0	< 40 or > 130<br>< 50 or > 120<br>50 ≤ HR ≤ 120<br>50 < HR ≤ 100			
Pain<br>At rest	3<br>2<br>1<br>0	Severe or VAS ≥ 70 mm<br>Moderate or 30 mm ≤ VAS < 70 mm<br>Mild or VAS < 30 mm<br>No pain or VAS = 0 mm			
Nausea	3<br>2<br>1<br>0	Severe<br>Moderate<br>mild<br>no nausea			
Motor skills	3<br>2<br>1<br>0	Not moving legs<br>Only able to move feet<br>Only able to move feet and bend knee<br>Moving both legs freely			
Diuresis	3<br>2<br>1<br>0	Anuria<br>0 < diuresis < 0.5 ml/kg/hour<br>0.5 ≤ diuresis < 1.0 ml/kg/hour<br>1.0 ≤ hourly diuresis OR no Urine catheter			
Temperature<br>(Tp) (°C)	3<br>2<br>1<br>0	Tp < 35.0<br>35.0 ≤ Tp < 35.5<br>35.5 ≤ Tp < 36.0<br>Tp ≥ 36.0			
Total	Sum	Total point score			

Proposal for general rules

All patients should at least be scored upon arrival to the post-anaesthesia care unit and twice at a 10 minute interval immediately prior to discharge from the post-anaesthesia care unit/dept. of anaesthesiology

Patients who fulfil the following conditions can be discharged from the post-anaesthesia care unit/department of anaesthesiology:

By a nurse if the following five criteria are met:

1) ASA < III OR ASA ≤ III [1)]
2) Uncomplicated surgical procedure
3) uncomplicated anaesthesia and postoperative stay in the post-anaesthesia unit
4) Blood loss ≤ 500 ml in patients with normal pre-operative haemoglobin
5) Adults (> 14 years) with point scores ≤ 1 and a total point score ≤ 4 at two consecutive measurements with at least 10 minute interval

[1)] Decided by the individual dept. of anaesthesiology

By a doctor if the following criteria are met:

1) ASA > III; prolonged (> 5 hours) or complicated per- or post-operative course in the post-anaesthesia care unit; blood loss > 500 ml or any point score ≥ 2 or total point score > 4 must be discharged by anaesthetist with interventions for the surgical ward staff
2) Patients with epidural catheter should be discharged by an anaesthetist with specification of dose rate, and stop date and time in accordance with current guidelines
