# Supplementary material for: Effect of intermediate care on mortality following emergency abdominal surgery. The InCare trial: study protocol, rationale and feasibility of a randomised multicentre trial
Source: Trials. 2013 Feb 2;14:37. doi: 10.1186/1745-6215-14-37 (PMC3575365; doi:10.1186/1745-6215-14-37)
Supplement: Additional file 3 — Surgeon - protocol-based round. [file 1745-6215-14-37-S3.pdf]

Patient ID:
Date:
Time:

Patient history (summary):
(if possible refer to previous notes)

Status:

Information: ☐ Patient is fully informed about the operation ☐ Relatives are fully informed about the operation
☐ Patient or relatives are partially informed: *(describe)*

☐ Not possible, *(give reason)*:

Abdomenal: Current nutritional needs are met: ☐ Yes ☐ No. Nausea: ☐ Yes ☐ No
Bandage is dry: ☐ Yes ☐ No, ____
Gastric-tube: ☐ No ☐ Yes, ________ml/24 hours. Bowel sounds: ☐ Yes ☐ No
Palpation: ____
Faeces: ☐ No ☐ Yes, ____
Flatus: ☐ Yes ☐ No
Drain: ☐ No ☐ Yes, *(describe)*

Extremities: Mobilisation status during the last 24 hours: ☐ Not mobilised ☐ To bedside ☐ To chair
☐ To a frame ☐ Walking with assistance ☐ Walking without assistance
Clinical signs of deep venues thrombosis (DVT): ☐ No ☐ Yes, *(describe)*_________________________
Is the patient on DVT prophylaxis: ☐ Yes ☐ No, *(why not)*__________________________________

Microbiological: Temperature:__________ WBC/CRP:______ /________
Antibiotics: ☐ Not given ☐ Empirically ☐ After culture ☐ Antibiotics not appropriate
Culture results:____
Other:

☐ See the intensivist's note on page:____

Para-clinical: Haemoglobin:_____ Coagulation:__________________ Diagnostic imaging/other:

Other:

Conclusion:
☐ Stable ☐ unstable, give reason(s):

Are there any postoperative complications: ☐ No ☐ Yes, *(describe)*:________________________________
Is there consensus with the intensivist: ☐ Yes ☐ Round pending ☐ No *(if NO, contact the attending intensivist)*

Some trial-sites use a dictation template with the same content because of technicalities

Plan:

CNS: Information about the operation:
- ☐ Not relevant
- ☐ The patient to be informed tomorrow
- ☐ Consultation with relatives
  Date______-_______Time:__________

Analgesia:
- ☐ See the intensivist's note on page:____
- ☐ Epidural analgesia is not indicated, continue planned pain treatment
- ☐ Continue epidural analgesia
  Discontinue on the:______-____
- ☐ Epidural infusion changed to:

Other:

Abdominal: Nutritional plan: *(within 24 hours)*
- ☐ Stated on a separate chart
- ☐ Continue ordinations
- ☐ No limits p.o ☐ Fluent nutrition ☐ Ice
- ☐ No intake p.o.
- ☐ Standard Protein fluid x _______ per day

____

Return to theatre:
- ☐ No ☐ Emergency ☐ scheduled operation,

date:______-______

Indication:

Other:
- ☐ Magnesia: Dose __________x__________

Drain:

☐ See the intensivist's note

Extremities: Thrombosis:
- ☐ Ultrasound of _____lower limb ☐_____________
- ☐ Continue DVT prophylaxis
- ☐ Discontinue DVT prophylaxis

Mobilisation:

☐ Not Indicated	☐ To bedside
☐ Chair ≥2 timer	☐ Chair ≥4 timer
☐ Chair ≥8 timer	☐ To a frame
☐ With frame x 3	☐ Walking x 3

Other:

Microbiological: Antibiotics:
- ☐ Not indicated
- ☐ Continue ordinations

____

____

Cultures:
____

☐ See the intensivist's note

Para-clinical: Blood samples:
- ☐ Daily haemoglobin, creatinine, sodium, potassium, bilirubin, platelets, WBC, CRP

____

Diagnostic imaging
____

☐ See the intensivist's note

Other: (incl. Respiratory/Cardiovascular/Renal)

*Title and name:*____________________________

Some trial-sites use a dictation template with the same content because of technicalities
