# Supplementary material for: Effect of intermediate care on mortality following emergency abdominal surgery. The InCare trial: study protocol, rationale and feasibility of a randomised multicentre trial
Source: Trials. 2013 Feb 2;14:37. doi: 10.1186/1745-6215-14-37 (PMC3575365; doi:10.1186/1745-6215-14-37)
Supplement: Additional file 4 — Intensivist - protocol-based round. [file 1745-6215-14-37-S4.pdf]

Patient ID:
Date:
Time:

Patient history (summary):
(if possible refer to previous notes)

Status:

CNS: Level of consciousness:___________________________________GCS:_____ VAS-score:_________

Pain treatment: ☐ Conventional ☐ Epidural analgesia_______________________________________

Trial consent status/other:

Respiratory: Supplemental oxygen:________ Oxygenation:_____ RR:_____ Stethoscopy:__________________

Blood-gas: pH:____, cause ☐ respiratory ☐ metabolic ☐ mix:______________________________

X-ray of thorax:____ ☐ None taken
Other:

Cardiovascular: BP:_______ Heart rhythm:________________ Heart rate:______ Peripheral perfusion:__________

Stethoscopy:_____________________________________ S_cVO_2:______ ☐ None taken

Volume status: ☐ normovolaemia ☐ hypovolaemia
Other:

Renal: Fluid balance:_______________ Sodium:______ Potassium:______ Creatinine:_________
Hourly diuresis:_______
Hydration status: ☐ Normohydration ☐ Dehydration ☐ Overhydration
Other:

Abdominal: Current nutritional needs are met: ☐ Yes ☐ No. Nausea: ☐ Yes ☐ No
Other:

☐ See the surgeon's note page:______

Microbiological: Temperature:__________ WBC/CRP: ______/________
Antibiotics: ☐ Not given ☐ Empirically ☐ After culture ☐ Antibiotics not appropriate
Culture results:____

Other:

☐ See the surgeon's note on page:____

Para-clinical: Haemoglobin:______ Coagulation:___________________ SOFA score:_____ SAPS II score:___

Diagnostic imaging/other:

Conclusion:
☐ Stable ☐ unstable, give reason(s):

Are there any postoperative complications: ☐ No ☐ Yes, *(describe)*:____
Is there consensus with the surgeon: ☐ Yes ☐ Round pending ☐ No *(if NO, contact the surgeon)*

Some trial-sites use a dictation template with the same content because of technicalities

Plan:

CNS:	Analgesia:	☐ Continue ordinations
		Other:_______________________________________
	Epidural analgesia:	☐ No ☐ Yes, ☐ discontinue date:_______- ______
		Other:_______________________________________
	Other:	
Respiratory:	Treatments goals:	☐ Oxygenation ≥ 94 % ☐ Contraindication
		Other:_______________________________________
		☐ 2 l oxygen during nights
	Pulmonary physiotherapy:	☐ Continue ordinations<br>☐ PEP-tube ☐ CPAP
		Physiotherapy frequency:_______________________
		Other:_______________________________________
	Other:	
Cardiovascular:	Treatment goals:	Blood pressure:_________ Heart rate:___________
		Diuresis:_______ Other:_______________________
	Monitoring level:	☐ Minimal monitoring level in the InCare-trial (cf. case report form page 20)<br>☐ BP/HR/SpO_2/RR ___ times per shift
	Other:	
Renal:	Fluid balance:	☐ Stated on a separate chart<br>☐ Not indicated, (reason)________________________
		Oral: _____________________________________ml
		IV.: ______________________________________ml
		IV.: ______________________________________ml
		☐ RBC transfusion:________________________units
Abdominal:	Nutritional plan:<br>*(Within 24 hours)*	☐ Stated on a separate chart<br>☐ Continue ordinations
		____
	Other:	
	☐ See surgeon's note page:_____	
Microbiological:	Antibiotics:	☐ Continue ordinations
		____
		____
	Cultures:	____
Para-clinical:	Blood samples:	☐ Daily haemoglobin, creatinine, sodium, potassium, bilirubin, platelets, WBC, CRP
		____
	Diagnostic imaging	____
	☐ See surgeon's note page:_____	
Others:		

Title and Name:______________________________

Some trial-sites use a dictation template with the same content because of technicalities
