# Supplementary material for: Effect of intermediate care on mortality following emergency abdominal surgery. The InCare trial: study protocol, rationale and feasibility of a randomised multicentre trial
Source: Trials. 2013 Feb 2;14:37. doi: 10.1186/1745-6215-14-37 (PMC3575365; doi:10.1186/1745-6215-14-37)
Supplement: Additional file 5 — Anaesthetist - discharge note. [file 1745-6215-14-37-S5.pdf]

Appendix 5: Anaesthetist — discharge note InCare-trial

Patient ID:
Date:
Time:

Patient history (summary):

(if possible refer to previous notes)

Status:

CNS: Level of consciousness:__________________________________GCS:_____ VAS-score:_________

Pain treatment: ☐ Conventional ☐ Epidural analgesia______________________________________

Trial consent status/other:

Respiratory: Supplemental oxygen:________Oxygenation:_____ RR:_____ Stethoscopy:___________________

Blood-gas: pH:____, cause ☐ respiratory ☐ metabolic ☐ mix:_____________________________

X-ray of thorax:____ ☐ None taken
Other:

Cardiovascular: BP:_______ Heart rhythm:________________ Heart rate:______ Peripheral perfusion:___________

Stethoscopy:_____________________________________ S_cVO_2:______ ☐ None taken

Volume status: ☐ normovolaemia ☐ hypovolaemia
Other:

Renal: Fluid balance:_______________ Sodium:______ Potassium:______ Creatinine:_________
Hourly diuresis:_______
Hydration status: ☐ Normohydration ☐ Dehydration ☐ Overhydration
Volume in: Out:

Fluid balance:__________

Abdominal: Nausea: ☐ Yes ☐ No. Bandage is dry: ☐ Yes ☐ No,______________________________________
Gastric-tube: ☐ No ☐ Yes, ________ml/24 hours. Bowel sounds: ☐ Yes ☐ No
Faeces: ☐ No ☐ Yes. Drain: ☐ No ☐ Yes, *(describe)*

Other:

☐ See the surgeon's note page:______

Microbiological: Temperature:__________ WBC/CRP:______/_____ Sepsis score:____ (cf. case report form page 17)
Antibiotics: ☐ Not given ☐ Empirically ☐ After culture ☐ Antibiotics not appropriate
Other:

Para-clinical: Haemoglobin:______ Coagulation:__________________ Diagnostic imaging/other:

Other:

Conclusion:

☐ Stable ☐ unstable, give reason(s):

Other:

Are there any postoperative complications: ☐ No ☐ Yes, *(describe)*:______________________________________

Some trial-sites use a dictation template with the same content because of technicalities

Plan:

CNS: Analgesia: ☐ Continue ordinations
Other:______________________________________

Epidural analgesia: ☐ No ☐ Yes, ☐ discontinue date_______- _______
Other:______________________________________

Other:

Respiratory: Treatments goals: ☐ Oxygenation ≥ 94 % ☐ Contraindication
Other:______________________________________
☐ 2 l oxygen during nights

Pulmonary physiotherapy: ☐ Continue ordinations
☐ PEP-tube ☐ CPAP
Physiotherapy frequency:______________________
Other:______________________________________

Other:

Cardiovascular: Monitoring level: ☐ BP/HR/SpO_2/RR ___ times per shift

Treatment goals: Blood pressure:_________ Hearth rate:___________
Diuresis:________Other:_______________________

Renal: Fluid balance – next 24 hours: Oral:______________________________________ml
IV.:_______________________________________ml
IV.:_______________________________________ml
☐ RBC transfusion:________________________units

Abdominal: Nutritional plan: ____
(Within 24 hours) ____
____

Other:

☐ See surgeon's note page:_____

Microbiological: Antibiotics: ☐ Continue ordinations
____
____

Cultures: ____

Para-clinical: Blood samples: ☐ Daily haemoglobin, creatinine, sodium, potassium, bilirubin, platelets, WBC, CRP
____

Diagnostic imaging ____

Trial consent: *(Remember name of legal representative if this option is used)*

Others:

Title and Name:_______________________________
